# Ankyrin Repeat and Kinase Domain Containing 1 Gene, and Addiction Vulnerability

**DOI:** 10.3390/ijms21072516

**Published:** 2020-04-04

**Authors:** Alejandra Koeneke, Guillermo Ponce, Johanna Troya-Balseca, Tomás Palomo, Janet Hoenicka

**Affiliations:** 1 Departamento de Psicología, Facultad de Ciencias Biomédicas, Universidad Europea Madrid, Villaviciosa de Odón, 28670 Madrid, Spain; alejandra.koeneke@gmail.com; 2 Departamento de Medicina Legal, Psiquiatría y Patología, Facultad de Medicina, Universidad Complutense de Madrid, 28040 Madrid, Spain; tomaspalomo@med.ucm.es; 3 Servicio de Psiquiatría, Hospital Universitario 12 de Octubre, Av. de Córdoba s/n, 28041 Madrid, Spain; gponcealfaro@gmail.com; 4 Laboratory of Neurogenetics and Molecular Medicine - IPER, Institut de Recerca Sant Joan de Déu, 08950 Barcelona, Spain; jtroyab@gmail.com; 5 CIBER de Salud Mental (CIBERSAM), Instituto de Salud Carlos III, 28029 Madrid, Spain

**Keywords:** ANKK1, *Taq*IA, DRD2, addictions, dopamine, neurodevelopment

## Abstract

The *Taq*IA single nucleotide variant (SNV) has been tested for association with addictions in a huge number of studies. *Taq*IA is located in the ankyrin repeat and kinase domain containing 1 gene (*ANKK1*) that codes for a receptor interacting protein kinase. *ANKK1* maps on the NTAD cluster along with the dopamine receptor D2 (*DRD2*), the tetratricopeptide repeat domain 12 (*TTC12*) and the neural cell adhesion molecule 1 (*NCAM1*) genes. The four genes have been associated with addictions, although *TTC12* and *ANKK1* showed the strongest associations. In silico and in vitro studies revealed that *ANKK1* is functionally related to the dopaminergic system, in particular with *DRD2*. In antisocial alcoholism, epistasis between *ANKK1* *Taq*IA and *DRD2* C957T SNVs has been described. This clinical finding has been supported by the study of *ANKK1* expression in peripheral blood mononuclear cells of alcoholic patients and controls. Regarding the ANKK1 protein, there is direct evidence of its location in adult and developing central nervous system. Together, these findings of the *ANKK1* gene and its protein suggest that the *Taq*IA SNV is a marker of brain differences, both in structure and in dopaminergic function, that increase individual risk to addiction development.

## 1. Introduction

Addictions are psychiatric disorders that modify the reward and learning systems of the brain [1], causing compulsive drug seeking and other maladaptive and destructive behaviors. Clinical studies have estimated that around 20%-40% of people who use substances of abuse will finally develop an addiction [2]. In the transition to addiction, not only the brain circuits of reward and motivation are involved, but also those of executive control and emotional processing [3]. Since gene-environment interactions underlie the functioning of brain circuits, individual differences in both genetics and environmental factors should synthesize the individual addiction vulnerability.

Family studies in twins [4] and the greater recurrence of addictions in affected families [5,6] support that the risk to develop an addiction is inherited. Addictions heritability values, which have been related to drug liability, range from 39% for hallucinogens to 72% for cocaine [7]. This heritability/liability correlation for particular drugs argues that a large component of what is inherited is related to individual variation in the neurobiological basis of addiction [7]. In animal models, it has been shown that the development of addictions is the result of the interaction between the degree of drug exposure and the individual vulnerable genotype [8]. As addiction develops in a small proportion of subjects, this finding also supports the genetic component of these disorders.

It has been proposed that addictions are inherited as complex diseases where a gene-environment interaction is required for their development [9]. During drug use, excessive dopamine signaling and epigenetic remodeling in the brain cause the shift to addictions [10]. In patients, a variety of environmental factors have been identified, such as early life trauma, altered family structure and social pressure. For example, it has been shown that deprivation of interactions and isolation during childhood increase the risk of developing an addiction [11,12]. On the genetic side, various loci and genes have been involved in addictions vulnerability including the gene Ankyrin repeat and kinase domain containing 1 gene (*ANKK1*). Specifically, the *ANKK1 Taq*IA single nucleotide variant (SNV) (rs1800497) is the most-studied genetic variant related to addictions [13,14,15,16]. The *ANKK1* gene encodes a putative kinase that is expressed in the developing and adult central nervous system [17,18]. Besides, several lines of evidence link this protein with the dopaminergic system [17,19,20]. However, the role of the *ANKK1* gene and its protein in the pathophysiology of addictions remain unknown.

In this review, we begin by presenting the actual status of the genetic association between the *ANKK1 Taq*IA SNV and alcoholism. As *ANKK1* is located near to the gene for the dopamine D2 receptor (*DRD2*), we also analyze the functional relationship of *ANKK1* and *DRD2* and have included experimental unpublished work of our laboratory that further supports this association in human peripheral blood mononuclear cells. We also proposed that *ANKK1/DRD2* relation explains, at least in part, the controversy about the *Taq*IA association to addictions and other dopamine-related phenotypes. In addition, here we included the genetic association studies between *ANKK1* locus haplotypes and different addictions. Finally, we summarize the progress that we have made in the biology of ANKK1 in cells and animal models. As a whole, we suggest that ANKK1 protein and its variations associate to addiction vulnerability on two levels: i) during development participating in signaling pathways involved in neurogenesis and ii) during adulthood modulating the dopaminergic function in the brain.

## 2. The Gene That Codes *ANKK1*, the *Taq*IA SNV, and Addictions

*ANKK1* spans around 14.38 Kb (NC_000011.10) on chromosome 11. This gene comprises at least 8 exons that code for a 765 amino acid protein that would act as a serine/threonine kinase [21]. Blum et al. [13] first reported the study of the relationship between the *ANKK1 Taq*IA SNV and alcoholism in Caucasians. *Taq*IA consists of a single Cytosine (C, A2 allele) to Thymine (T, A1 allele) change in the *ANKK1* gene. Since then, several groups have replicated the association between alcoholism and this genetic variant [22,23,24,25,26,27,28,29,30], although other authors have not found this association [31,32,33,34,35,36,37,38,39,40,41,42,43,44,45,46,47,48,49]. This inconsistency has been resolved by four meta-analyses including a large number of Caucasian alcoholic patients and controls. Two meta-analyses of the *Taq*IA confirmed an association between the A1 allele and alcoholism [50,51]. Another study of common SNVs underlying drug addiction found that *Taq*IA is the third most significant genetic variation associated with alcohol-related phenotypes [52]. The most recent study meta-analyzed 62 reports followed by a meta-regression to identify hidden confounders [53]. The pooled odds ratio (OR) not only estimates that the *Taq*IA A1 allele increases the risk of alcohol dependence but also claims that the relationship between this SNV and alcoholism, is one of the largest effects ever observed for a polymorphic genetic variant in psychiatry (OR, 1.23; *p* < 0.001). The authors state that the association is not attributable to dopamine receptor D2 (D2R) functional differences [53].

The *Taq*IA A1 allele has been also associated with severe alcoholism and non-adaptive behavior [54]. Moreover, the genotype A1+ (heterozygous and homozygous for the *Taq*IA A1 allele) and cannabinoid genetic variants have an additive effect on the Hare Psychopathy Checklist-Revised (PCL-R) scores in severe alcoholic patients [55]. These findings suggest that there is a shared genetic liability for addictions and antisocial disorders.

*ANKK1* has also been studied in other substance use disorders [15,56]. For instance, there are two meta-analyses of the association between the *Taq*IA and smoking behavior, but with conflicting results [57,58]. The *Taq*IA A1 allele has also been associated with opioids addiction [59,60,61,62] and with a lower response to methadone treatment in addicted patients [59,63,64,65]. A recent meta-analysis shows that *Taq*IA was significantly associated with increased risk of opioid dependence under homozygote, dominant, and recessive genetic models (homozygote: OR = 1.546, 95%CI = 1.279–1.87; dominant: OR = 1.265, 95%CI = 1.055–1.516; recessive: OR = 1.409, 95%CI = 1.182–1.680) [66]. Therefore, *ANKK1 Taq*IA SNV is still a current genetic marker for addictive disorders.

## 3. The Functional Relationship of *ANKK1* and *DRD2*

All drugs mediate their reinforcing properties, causing a large and fast release of dopamine in limbic regions [67]. The chronic use of drugs may raise the thresholds required for dopaminergic neurons activation and dopamine signaling in response to salient stimuli. Indeed, patients with addictions have deficits in brain dopamine function in the orbitofrontal cortex, the cingulate gyrus and the dorsolateral prefrontal cortex [68]. At the same time, conditioning triggered by drugs leads to enhanced dopamine signaling in striatal and prefrontal areas when the patients are exposed to conditioned drug-associated cues. All these changes result in the loss of control and compulsive drug intake characteristic of addictions [68]. Overall, dopamine deregulation associated with drug use causes the dysfunction of the reward pathway. Therefore, dopamine is a key neurotransmitter of the pathophysiology of addictions and the genes involved in dopaminergic transmission could mediate the risk of these disorders.

Going back to the gene that encodes *ANKK1*, we have found that this gene is linked to the dopaminergic system. In human cellular models, we showed that the *ANKK1* gene and protein expression levels are affected by apomorphine (APO) [17,19], a non-selective dopaminergic agonist which acts as a partial agonist of the dopamine receptor D1-like subclass (D1R-like) and a full agonist of the dopamine receptor D2-like subclass (D2R-like). In HEK293 cells, the *Taq*IA SNV had a significant impact on the expression of the recombinant human ANKK1 both at basal levels and when stimulated with APO [19]. In adult mouse brain, *ANKK1* mRNA expression was oppositely regulated by D1R-like and D2R-like activation [20]. Specifically, the treatment of mice with the D1R-like selective agonist SKF38393 or APO causes an *Ankk1* gene up-regulation. SKF38393 binds to D1R and D5R that signal via G proteins through protein kinase A (PKA) and phospholipase C (PLC) pathways, respectively [69]. In contrast, treatment with the D3R agonist 7-OH-DPAT, which induces the activation of phospholipase D (PLD) [70], causes a strong *Ankk1* downregulation. PLD regulates downstream effectors, such as protein phosphatase 1 (PP1) that controls transcriptional events that are relevant for memory formation and synaptic plasticity in the brain [71,72]. Taken together, these findings suggest that *Ankk1* expression could be the result of a net effect of competing pathways of different dopamine receptors.

*In silico* studies revealed a tight relation between *ANKK1* and *DRD2* genes. On the one hand, *ANKK1* is adjacent to the *DRD2* gene [21], shares haplotypic blocks and their promoters have cis elements in common for transcriptional regulation [17,73]. Additionally, *Taq*IA is in linkage disequilibrium with *DRD2* variants that regulate the expression of long and short D2R isoforms [73,74], and modulates D2R distribution in the ventral striatum [75]. *Taq*IA A1 allele has also been associated with increased activity of striatal L-amino acid decarboxylase, the final enzyme for dopamine synthesis [76].

The *ANKK1/DRD2 locus* has another SNV associated with brain dopaminergic functions that consists of a C to T synonymous change within exon 7 of the *DRD2* gene [77]. This SNV named C957T (rs6277) has a marked impact on D2R availability [78] and it is also a marker of regulatory regions at the 5′ UTR of the *ANKK1* gene [73]. In a sample of alcoholic patients, we found an epistatic effect of the *ANKK1 Taq*IA and *DRD2* C957T SNVs on the expression of antisocial traits [79]. Specifically, the risk genotypes A1+ for the *ANKK1 Taq*IA and CC for *DRD2* C957T SNVs were associated with comorbid dissocial personality disorder and high scores on the PCL-R [79]. Moreover, there were no differences between individuals who did not carry any of the risk genotypes and those that carried only one of them, either A1+ for *Taq*IA or CC for C957T. Therefore, it seems that *ANKK1* and *DRD2* genes could somehow interact with the dopamine signaling pathways or act in a coordinated manner, affecting individual vulnerability to both addictions and antisocial disorder.

The *ANKK1/DRD2 locus* has also been related to cognitive and emotional processes [73,80] that could be associated with risk for developing addictions. A recent systematic review and meta-analysis evaluated the association of *ANKK1 Taq*IA and *DRD2* C957T SNVs in the performance of the executive functions in healthy adults [81]. Three main domains of executive functions were analyzed: response inhibition, cognitive flexibility and working memory. Regarding *Taq*IA, previous reports found that A1 allele carriers have been related to worse working memory performance [82,83,84], or with less cognitive flexibility [82,85,86]. However, after meta-analysis, the signification was lost [81]. C957T SNV’s previous positive association with working memory function [87,88,89], and response inhibition [87,88,90] were also negative after a meta-analysis, while cognitive flexibility was still associated. Some of these inconsistencies could be attributed to both the heterogeneity of the executive function tasks used and the variability of the populations investigated [81]. Another source of heterogeneity, which might have contributed to the lack of significant findings, could be the differences in the grouping of the genotypes between studies. While some studies compared CT vs. TT vs. CC genotypes [90], others compared CC vs. CT/TT [55]. However, given the functional relationship between *ANKK1 Taq*IA and *DRD2* C957T SNVs in the dopaminergic system, the role of these genetic variants and their possible interactions upon the executive function or other cognitive processes should not be excluded.

**Figure 1:**
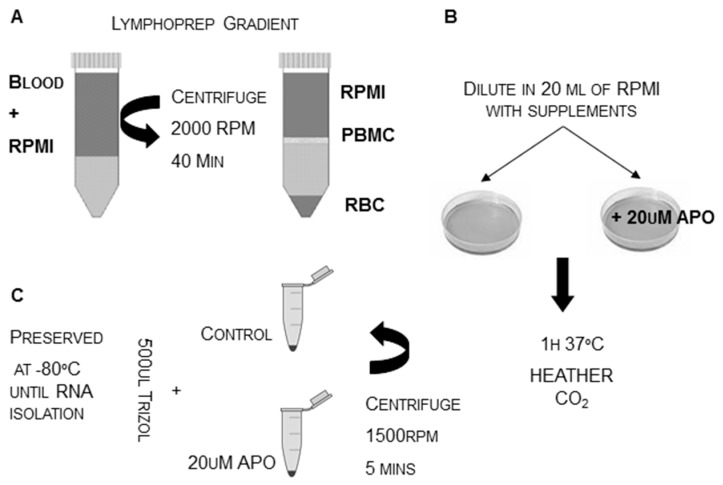
Protocol to obtain human peripheral blood mononuclear cells (PBMCs). (**A**) For each sample PBMCs were isolated in a Lymphoprep gradient, (**B**) diluted in 20 mL of RPMI and divided into two plates. One plate corresponds to basal conditions (baseline) and the other was treated with APO. (**C**) After the PBMCs pellets were isolated by centrifugation at 1500 rpm. RNA extraction was performed using conventional protocols. RPMI: Roswell Park Memorial Institute medium; RBC: red blood cells; APO: apomorphine.

To gain further biological insight into the functional relationship among *ANKK1*, *DRD2 and* the dopaminergic system, we have studied *ANKK1* expression in human peripheral blood mononuclear cells (PBMCs) in healthy controls and detoxified alcoholic patients. PBMCs were isolated by a density gradient medium (Lymphoprep) and treated with APO, as shown in Figure 1. PBMCs express dopamine receptors and dopamine transporters and also synthesize dopamine through tyrosine hydroxylase-dependent mechanisms [91,92]. PBMCs total RNA from patients with alcoholism (n: 24) and controls (n: 17) was extracted using TRIZOL. PolyT primers were used for the RT reaction (Maxima^®^ First Strand cDNA Synthesis Kit, Fermentas) and all quantitative real-time PCR reactions were carried out in triplicate on a 7500 Fast Real-Time PCR System (Applied Biosystems, Foster City, California, USA). RT-PCRs were performed as previously described [17].

**Figure 2:**
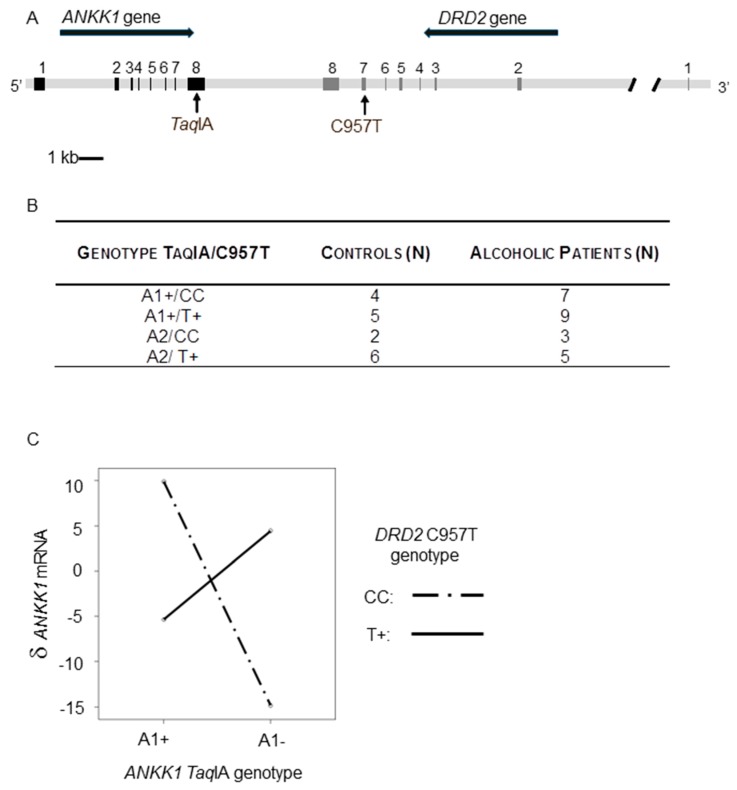
*ANKK1* and *DRD2* genes interaction effect on *ANKK1* expression levels of human PBMCs after apomorphine (APO) treatment. (**A**) *ANKK1/DRD2* locus showing the location of *ANKK1 Taq*IA and *DRD2* C957T SNVs. Black and grey boxes are *ANKK1* and *DRD2* exons, respectively. (**B**) *ANKK1 Taq*IA genotypes: A1+ (A1 allele homozygous and heterozygous) and A1- (homozygous for the A2 allele) and, *DRD2* C957T genotypes: CC (C allele homozygous) and T+ (homozygous for the T allele and heterozygous) in a clinical series of alcoholic patients and control individuals. (**C**) δ *ANKK1* mRNA: *ANKK1* mRNA level differences after APO treatment.

We evaluated *ANKK1* and *DRD2* interaction in PBMCs using a regression model including the genotypes for the two genes (*ANKK1*: *Taq*IA and *DRD2*: C957T) and, *ANKK1* expression differences (δ: mRNA levels after APO treatment—mRNA levels at baseline) as a quantitative dependent variable. We found a significant epistatic effect of the *ANKK1 Taq*IA and *DRD2* C957T SNVs upon *ANKK1* mRNA δ in healthy controls (F: 29.4, *p* < 0.0001), alcoholic patients (F: 11.6, *p* < 0.003) and the total sample (F: 35.2, *p* < 0.0001). Figure 2 shows that the interaction of the genotype of any of the genes with the variant of the other gene has a radically different significant effect upon *ANKK1* δ. In A1 + carriers, the CC genotype of *DRD2* is associated with a significant increase in *ANKK1* δ. On the contrary, *ANKK1* δ is decreased in A1-/CC genotype subgroup. Therefore, *ANKK1* δ associated with the CC genotype in A1 individuals is opposite to that observed among carriers of the A1+/CC genotype.

These findings in healthy controls and detoxified alcoholic patients not only support that ANKK1 function is closely related to dopaminergic activity, but also suggest coordination between ANKK1 and D2R. We propose that ANKK1 is involved in addictions by modulating the brain dopaminergic response at least in the dopaminergic neurons and the striatum where the D2R receptor has the highest density. Since *Taq*IA is tagging both *ANKK1* and *DRD2* functional gene variants, our in vitro results in human samples represent a broader scenario to help understanding neuropsychiatric disorders associated with this SNV.

## 4. The Locus of the Gene *ANKK1* and Addictions

*ANKK1* gene maps to chromosome 11q22-q23 (chr11:11338038-113400418; GRCh38/hg38) in a 521-kb conserved gene cluster among vertebrates [93], along with the neural cell adhesion molecule 1 (*NCAM1*), *DRD2* and the tetratricopeptide repeat domain 12 gene (*TTC12*) genes. Extensive genotyping of the *NCAM-TTC12-ANKK1-DRD2* (NTAD) cluster shows that substance abuse is associated with variants of the 4 genes.

The NTAD cluster has been studied in alcoholism in family-based and case-control designs. One study found associations in exon 12-intron 13 of *NCAM1*, exon 3 of *TTC12*, and exons 2 and 5 of *ANKK1* haplotypes but not with *DRD2* [94]. Besides, haplotypes in *TTC12* exon 3, *NCAM1* exon 12, and the two 3′ ends of *ANKK1* and *DRD2* genes, including the *Taq*IA SNV (the variant named A7 in this study) have been linked to comorbid alcohol and drug dependence in European-Americans [95]. Another study analyzed the involvement of *DRD2* and *ANKK1* in 219 Caucasian families with alcoholism, using data collected as a part of the Collaborative Study on the Genetics of Alcoholism (COGA) [96]. The strongest association was found between the subset of alcoholics with antisocial personality disorder and the *ANKK1* SNV rs4938012 that is located in the 5′ block of the *ANKK1* gene.

The NTAD cluster has been studied in other addictions. For instance, in nicotine addiction, family-based association studies in Caucasian and African American affected sib-pairs, showed strong and consistent evidence of an association with the *TTC12* (rs2303380, rs2282511) and *ANKK1* (rs4938012, rs4938013, rs4938015) genes [97]. Other studies of NTAD cluster identified in the African-American and European-Americans/African-Americans pooled sample the association between the *ANKK1* rs2734849 SNV and nicotine dependence [98]. Recently, a study of 268 African American daily smokers reported that primary dependence motives (PDM) are intermediate phenotypes associated to the NTAD cluster in nicotine dependence [99]. Specifically, the authors found significant associations between automaticity motives and both *TTC12* (rs2282511)/*ANKK1* (rs877138) and *ANKK1* (rs17115439/rs4938013) haplotypes. These SNVs were also associated with automaticity in a sample of European-Americans smokers [100]. In a large male Chinese Han smokers sample (n: 2.616 patients), the study of the NTAD cluster identified two missense *ANKK1* SNVs (rs11604671, rs2734849) and one intronic *DRD2* SNV (rs4648317) associated with nicotine addictions [101]. The NTAD cluster has also been studied in 1459 Australian heroin-dependent cases receiving opioid replacement therapy. In this sample, evidence of an association between opioids addiction and *ANKK1* (rs877138, rs4938013) and *TTC12* (rs7130431) was found. This finding was replicated when comparing illicit drug-dependent and non-dependent neighborhood controls [102].

The genes that comprise the NTAD cluster show a conserved synteny and neighborhood in 22 vertebrate species using available databases [93]. In patients with addictions and healthy controls, the study of haplotypes within this cluster shows high linkage disequilibrium, haplotypes shared by several genes, and some SNVs in one gene that are also markers of another gene [73]. From a genetic perspective, these findings suggest the functional coordination of the proteins coded by genes of this cluster. Indeed, a genomic cluster is defined by a group of genes sharing genomic location and belonging to a common category, such as involvement in the same pathway or function. *NCAM1* and *ANKK1* have been implicated in neurogenesis [17,18,103] and the *ANKK1 Taq*IA SNV has been associated with the morphology of the midbrain [104], the anterior cingulate cortex [105] and the caudate volume [106] in humans. Since *NCAM1* and *ANKK1* are expressed during development, the NTAD cluster association with addictions would point to inherited structural differences in brain circuits that are involved in addictions. These differences would be responsible for the risk of addictions associated with the NTAD cluster, especially with *ANKK1* SNVs.

## 5. ANKK1 Protein, Brain Structure, and Addiction

To date, more than one thousand *Taq*IA genetic association studies in clinical samples and healthy controls have been reported in the literature, most of which assume that this polymorphism is a marker of the *DRD2* gene expression. However, as mentioned above, the *DRD2* gene is located nearby the *ANKK1* gene, which is also linked to dopaminergic function in the brain [20,76]. Therefore, there is a need to incorporate what we know today about the ANKK1 biology for a broader interpretation of the *Taq*IA-association genetic phenomenon. Indeed, in 2008 it had been proposed that the absence of data about ANKK1 biology is a difficulty when interpreting the link between the *Taq*IA and the associated phenotypes [107,108].

In 2010, our group provided the first direct evidence of ANKK1 location in adult and developing central nervous system (CNS) in astroglial cells [17]. In silico studies of *ANKK1* cDNA clones and predicted peptides in *Homo sapiens*, *Mus musculus*, and *Rattus norvegicus* showed that there are at least 3 isoforms. The three isoforms are (i) ANKK1 full-length that includes a RIP kinase and ankyrin repeat domains (84 Kd), (ii) ANKK1-kinase with only the RIP kinase domain (~54 Kd), and (iii) ANKK1-ankyrin with only the ankyrin repeat domain (~66 Kd) [17]. The *Taq*IA SNV would affect all of them. *Taq*IA causes the change of glutamic acid to lysine at residue 713 (E713K) in the ankyrin repeat domain [21] and it is in linkage disequilibrium with the change alanine to threonine in position 239 (A239T) in the kinase domain [19]. Since A239T could modify the kinase activity of ANKK1 [19], *Taq*IA is a marker of both the kinase domain and the ankyrin repeat domain of ANKK1 [17,19].

**Figure 3:**
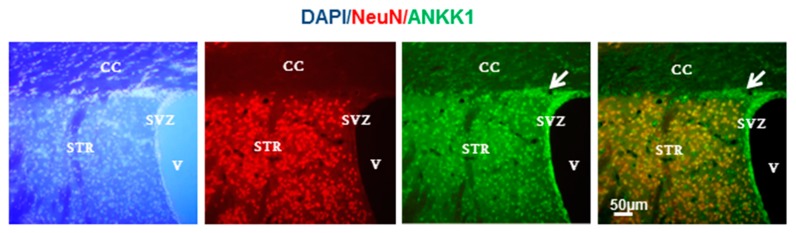
ANKK1 is expressed in post-mitotic neurons and also in adult neural stem cells and migrating neuroblasts of the adult mouse brain [18]. Immunostaining of ANKK1 and NeuN (marker of mature neurons) has been performed as previously described [18]. The white arrow indicates migrating neural precursors. CC: corpus callosum, STR: Striatum, SVZ: Subventricular zone, V: ventricle. Images were taken from confocal optical sections that are representative for the group averages. Scale bars: 50 μm.

Later investigations of the biology of ANKK1 unveiled that *ANKK1* mRNA and protein isoforms are expressed in adult and developing CNS, not only in astroglial cells, but also in the nuclei of postmitotic neurons and neural precursors from neurogenic niches in human and mouse brain [18] (Figure 3). We also found that ANKK1 has an impact on the cell cycle that is affected by *ANKK1* polymorphisms and APO treatment [18]. Additionally, the expression of *ANKK1* is not restricted to the CNS, but extends to the myogenic lineage [109]. We observed ANKK1 in migrating myoblasts and satellite cells, which are muscle precursors. Thus, ANKK1 is located during development in migrating precursors of neurons and myotubes, suggesting that this protein participates in signaling pathways that control cell migration. Since *NCAM1* is also expressed in neural and myogenic precursors [103,110,111], *ANKK1* and *NCAM1* genes could play a coordinated role during development.

ANKK1, also known as RIP5, belongs to the RIP serine–threonine kinase family, which regulates and induces both intracellular inflammatory signaling pathways or, apoptotic or necrotic cell death [112,113]. The RIP family participates in the differentiation of a broad variety of cell types and tissues [114]: RIP2 and ANKK1 in myogenic differentiation [109,115], RIP4 (homologous to ANKK1 at both the N-terminal kinase domain and the C-terminal ankyrin repeats) in epidermal morphogenesis during development and cutaneous wound repair [116] and, RIP7 and ANKK1 in neuronal differentiation [17,18,117]. Altogether, these data are in agreement for a relevant role of ANKK1 during development. Particularly, ANKK1 cell biology findings in the CNS assign a potential role for inheritance brain structure in *Taq*IA-associated phenotypes.

## 6. Conclusion and Future Considerations

Here, we have reviewed both the genetic evidence of the *ANKK1 Taq*IA SNV association with addictions and the biological evidence of the function of ANKK1.

**Figure 4:**
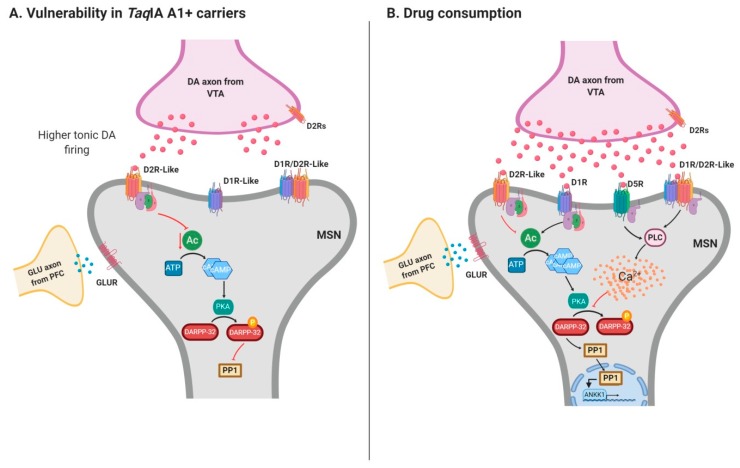
Proposed model for the relationship of ANKK1, the dopaminergic system in the mesolimbic brain and addiction. (**A**). The vulnerability of *ANKK1* A1+ genotype carriers and tonic dopamine firing, (**B**). Drug consumption, phasic DA firing and *ANKK1* expression. DA: dopamine (rose dots); VTA: ventral tegmental area; D2R: DA receptor D2; MSN medium spiny neurons; AC: adenyl cyclase; D1R: DA receptor 1; PLC: Phospholipase C beta; D5R: DA receptor 5; D1R-like/D2R –like: DA hetero-oligomers; cAMP: cyclic AMP; Ca^2+^: calcium (yellow dots); DARPP-32: DA- and cAMP-regulated phosphoprotein, Mr 32,000; PP1: protein phosphatase 1 GLUR: glutamatergic receptor. Created with BioRender.

We hypothesize that ANKK1 is involved in the pathophysiology of the *Taq*IA-associated phenotypes during development, participating in neurogenesis and during adulthood by modulating the dopaminergic function (Figure 4). *ANKK1* SNVs may be related to subtle cytoarchitecture changes in the brain that increased the risk of developing an addiction. Moreover, since *ANKK1* is linked to the dopaminergic function, it is plausible that *ANKK1* genetic variants also confer an additional risk in vulnerable individuals during drug use, by differences in their interaction with the *DRD2* gene and with other components of the dopaminergic system. Individuals that carry *ANKK1* A1+ genotype would have structural differences compared to A1 – genotype [18]. These structural differences related to *Taq*IA would cause increased dopamine biosynthesis [76] in the dopaminergic neurons of the ventral tegmental area (VTA), which results in higher tonic dopamine firing. Tonic dopamine stimulates D2R-like inhibitory signaling pathways in medium spiny neurons (MSN) of the striatum. Prior to drug use, high levels of dopamine in the brain have been linked to both increased impulsive reward-seeking and dopamine responsivity, which may be risk factors for addiction development [118] (Figure 4A). When consumption of a drug occurs, phasic dopamine neuronal firing stimulates both adenyl cyclase (AC) at the D1R and PLC-beta at the D5R or D1R-like/D2R-like hetero-oligomers, raising the levels of cyclic AMP (cAMP) or calcium, respectively. The dopamine- and cAMP-regulated phosphoprotein, Mr 32,000 (DARPP-32) plays a central role in integrating cAMP and calcium signaling in MSNs. The increase in intracellular calcium results in DARPP-32 dephosphorylation and nuclear PP1 activity [119], which would control *ANKK1* expression (Figure 4B). The carriers of *ANKK1* A1+ genotype with higher tonic levels of dopamine may also have differences in phasic dopamine firing that could contribute to the transition from drug use to the emergence of an addictive disorder.

More research is needed to decipher ANKK1 biology and its participation in the vulnerability to addictions. The development of animal models to study ANKK1 and their in-depth phenotype will help to understand the underlying biology of the *Taq*IA genetic association with so many phenotypes. It is also likely that multiple pathophysiological mechanisms may contribute to the association of this well-studied SNV. Further investigation of ANKK1 biology, in cellular and animal models, will provide actionable biological insight about the pathophysiology and future therapies of addictions.
